# Supplementary material for: NeKo: A tool for automatic network construction from prior knowledge
Source: PLoS Comput Biol. 2025 Sep 16;21(9):e1013300. doi: 10.1371/journal.pcbi.1013300 (PMC12456767; doi:10.1371/journal.pcbi.1013300)
Supplement: S1 Text — The code and figures used for both the main article and the supplementary materials can be found at the following github page: https://github.com/sysbio-curie/NeKo_Supp_Mat. (PDF) [file pcbi.1013300.s001.pdf]

Supplementary Information - NeKo: a tool for automatic network construction from prior knowledge

Ruscone et al.

August 17, 2025

This document contains additional details and analysis that complement the work presented in the main text. The code and figures used for both the main article and the supplementary materials can be found at the following github page: `https://github.com/sysbio-curie/NeKo_Supp_Mat`.

Contents

S1 Package availability and composition 1

S2 Troubleshooting 2

S3 Link between Python functions and NeKo strategies 3

S4 How to integrate a new database in the Neko's Universe class 3

S5 Additional analysis on Use case 1 - differences between Signor and HuRI 5

S6 Additional analysis on Use case 2 - Topological differences between Flobak et al. and NeKo 11

S7 Benchmark 12

S1 Package availability and composition

The source code of NeKo is available on GitHub at the following page: `https://github.com/sysbio-curie/Neko`. In addition to the modules represented in Fig A, the package includes a folder "data" with dedicated functions to import Signor (requires a separate download) and the PhosphositePlus databases. NeKo can be installed via:

```
pip install nekomata
```

or directly by source using:

```
git clone https://github.com/sysbio-curie/Neko
cd Neko
pip install .
pip install -r requirements.txt
```

In addition, we included a tutorial made with Jupyter notebooks to showcase NeKo's main features and showing how to use the classes that compose the package. The tutorial is available at the following link: `https://sysbio-curie.github.io/Neko/`. The notebooks are also included in the folder "notebooks" on github (`https://github.com/sysbio-curie/Neko/tree/main/notebooks`) ready to run.

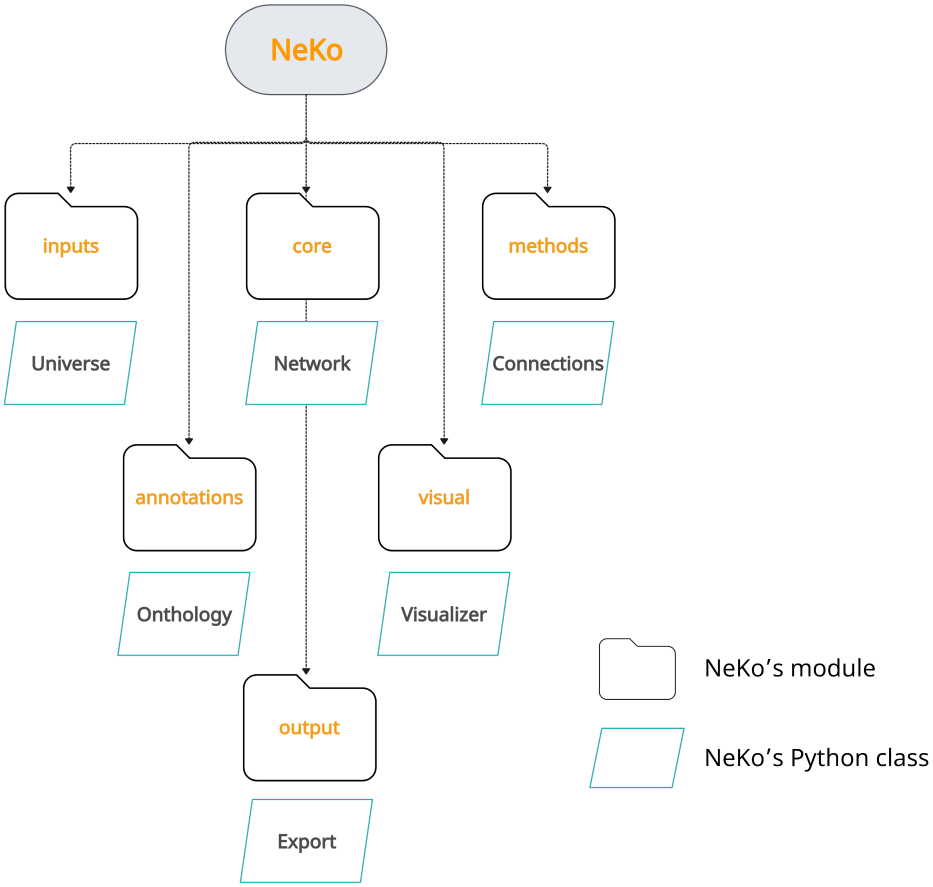

Figure A. Composition of the NeKo package. Each package module (represented by a folder) contains the classes that compose the package. The module "core" contains the `Network` class, while "inputs" contains the `Universe`. The "methods" module contains the `Connections` class. The "annotations", "visual" and "output" contain respectively the `Onthology`, `Visualizer`, and `Export` classes.

S2 Troubleshooting

Despite the package installation has been extensively tested on multiple platform, it could be possible to encounter some errors during the installation of NeKo. For now, the most common ones are related to Graphviz and Pypath. Upon importing NeKo in a Jupyter Notebook, in particular the module `NetworkVisualizer`, an error regarding the Graphviz installation could arise. In this case, make sure to have pygraphviz installed in your conda/mamba environment (it is a NeKo requirement, so it should be automatically downloaded when installing NeKo). Once verified, make sure Graphviz is installed in your machine, outside the environemnt. You can install Graphviz on Linux system with the following command:

```
sudo apt-get install python3-dev graphviz libgraphviz-dev
```

or on Mac system:

```
brew install python3-dev graphviz libgraphviz-dev
```

For more details visit: `https://graphviz.org/download/`.

The Pypath package is a component of NeKo that handles the translation of identifiers from Genesymbol to Uniprot. When importing NeKo for the first time, Pypath will create a folder (usually in your home, /home) containing some cache files necessary for its usage. During the process of downloading these files, some errors could occur, preventing Pypath from reading them correctly. This

will rise some errors in the execution of the basic NeKo operation. One possible solution is to locate and empty the cache folder, forcing a new download of those files at the next NeKo import.

More information can be found in the Pypath book: `https://pypath.omnipathdb.org/notebooks/manual.html`.

Other errors concerning the NeKo package can be reported in the Issues page of the NeKo GitHub page: `https://github.com/sysbio-curie/Neko/issues`.

S3 Link between Python functions and NeKo strategies

In the network class the user can find different functions that allows to run the strategies explained in the main text (see section "Connection Strategies" of the main text). Table A illustrates which function correspond to which strategy.

Each function can be adapted to the user's preferences by adjusting the parameters. We report in the following list a description of the parameters that characterize the function complete_connection, which implements the Reciprocal Pathway Extender strategy.

- Network.complete_connection
  - maxlen: The maximum length of the paths to be searched for. Default is 2.
  - algorithm: The search algorithm to be used. It can be 'bfs' (Breadth-First Search) or 'dfs' (Depth-First Search).
  - minimal: A boolean flag indicating whether to update the network every time a new path is added in the nodes iteration. If set to False, it will look for paths between nodes pair in the Universe ignoring exisiting paths in the network. Default is True.
  - only_signed: A boolean flag indicating whether to filter unsigned paths. Default is False.
  - consensus: A boolean flag indicating whether to check for consensus among references. Default is False.
  - connect_with_bias: A boolean flag that controls whether new nodes are immediately connected to their neighbors when first added to the network. The default is False. If set to True, each time a new node is introduced via a new path, the function attempts to connect the node to its immediate neighbors that are already in the network. This can save time by reducing the need to search for paths between pairs of nodes later. However, using this option comes with a trade-off. While it improves efficiency by skipping iterative searches, it also risks missing potential longer or alternative paths that might have been discovered through a more exhaustive search. Therefore, the choice depends on whether you prioritize faster computation (with possible path omissions) or a more thorough pathfinding process (which may take longer but captures all possible connections).

The API of NeKo (`https://sysbio-curie.github.io/Neko/api.html`) contains a description of the parameters of all the functions included in the Network class.

S4 How to integrate a new database in the Neko's Universe class

NeKo by default implements the Omnipath database [1] in the Universe class. Omnipath can be initialized directly from the Network class:

```
neko_network = Network(list_of_nodes, resources = 'omnipath')
```

In order to use a different database for the Universe, it is necessary to provide it directly to the Universe class, using the appropriate functions. As example, we report the steps necessary to integrate Signor and Huri in the Universe class. The same example is reported in the NeKo documentation.

Strategy	Python Function
Reciprocal Pathway Extender	*Network.complete_connection* and *Network.connect_subgroup*
Iterative Neighbor Expansion	*Network.connect_radially*
Regulatory Cascade Explorer	*Network.connect_to_upstream_nodes*
Module Connection Mapper	*Network.connect_component*
Phenotype Integration and Network Connectivity	*Network.connect_genes_to_phenotype*

Table A. The table illustrates which python functions implement the corresponding strategy.

- Signor
  - Download the file from the Signor webpage: `https://signor.uniroma2.it/downloads.php`. Select "Complete Dataset", chose the organism "Human" and select the ".tsv" format.
  - Place the downloaded file in your NeKo project folder.
  - In a Jupyter notebook, import the NeKo modules:

```
from neko.core.network import Network
from neko.inputs import Universe, signor
```

  - Once imported the modules, create the Universe providing the path of the .tsv file downloaded previously. Build the database.

```
resources = Universe()
resources = signor("../neko/_data/signor_db.tsv")
resources.build()
```

  - Create the Network object specifying the newly created Universe as resources:

```
neko_network = Network(list_of_nodes, resources = resources.interactions)
```

- Huri
  - Download one or more of the files from the Huri webpage: `http://www.interactome-atlas.org/download`. For the use cases shown in the main text, we selected all the files from Luck et al [2] but for this example we will focus on the file "HI-union".
  - Once downloaded the file (in .tsv format), move it in your Neko project folder.
  - The file contains just two columns with the Ensamble ids of the proteins. In this case our Universe is unsigned. In order to use this database, we need to translate the identifiers from Ensamble to Uniprot. To do this, we can use the Translator module within NeKo.
  - In a Jupyter notebook, import the translator:

```
from neko.inputs.db_translator import IDTranslator
```

  - Translate al the identifiers using the following function:

```
translator = IDTranslator('HI-union.tsv', 'genes_translated.csv', 'Ensembl', 'UniProtKB-Swiss-
    Prot', has_header=False, input_columns=['source', 'target'], processes=12)
translator.run()
```

  - The function will start traslating the unique identifiers of the database. This process can take up to one hour, but at each step, a pickle file will be created to save the progress of the translation. In case the translation stops, it can be resumed from the latest saved pickle file. A new file called "gene_translated.csv" will be created. This file will be used to create a NeKo Universe.

- The file contains just two columns and no information about the sign of the interaction. We can use the function implemented in the Universe class to adapt it to the NeKo structure.
- We start by creating the Universe. Next, we define a dictionary, specifying which column we want to use as "source" and which one we want to use as "target".

```
resources = Universe()
mapping = {"source_UniProtKB-Swiss-Prot": "source", "target_UniProtKB-Swiss-Prot": "target"}
resources.add_resources(huri, columns=mapping,  reset_index=True)
```

- Finally we build the Universe and create the Network object specifyin the newly created Universe as resources:

```
resources.build()
neko_network = Network(list_of_nodes, resources = resources.interactions)
```

More examples on how to build a custom Universe can be found in the NeKo documentation, in the notebook "Build network using user-defined resources".

S5 Additional analysis on Use case 1 - differences between Signor and HuRI

An important aspect to take into account when using NeKo, is the impact of the chosen Universe (the database containing the interactions to mine) on the final network topology. Databases like Omnipath, contain a high number of interaction from multiple sources. but this can lead to introduce some off-topic interactions in the network (i.e. all the interactions that are un-related to the initial biological question).

To highlight the differences that arise from using different databases, we have conducted the same analysis proposed in the main text for Use Case 1 (which was based on the HuRi database) starting from the Signor database.

While HuRI focuses on physical protein-protein interactions resulted from experimental techniques, Signor 3.0 focuses on the causal relationships between proteins.

The advantage of using databases like Signor is that the type of interaction is known including the directionality, resulting in directed and signed networks. On the other hand, many interactions in Signor have been reported both as activation and inhibition, depending on the context. In Neko, given a specific context, the user needs to further refine the template model by manually selecting the sign of the "bimodal" interaction. Fig C report the networks for each subgroup built from both Signor (directed and signed) and HuRI (directed but not signed). The networks built starting from Signor contains both positive (green arrows) and negative (red arrows) interactions. Moreover, when possible (according to the database annotations), NeKo can recognize complex formation (blue arrows). Given the intrinsic properties of PPI networks, networks inferred on HuRi database are unsigned and undirected.

Fig 4 (main text) and Fig D show the networks inferred based on hallmark genes characterizing four different subgroups, which were manually selected from [3] and [4], using Huri and Signor, respectively HuRi and Signor do not contain the same number of nodes. Some genes available for HuRi were not available for Signor. This point introduces an intrinsic bias in the construction of the networks using different databases determining overall, a lower number of edges and nodes in the network created with Signor (Fig D B) and a higher one when using HuRi (Fig. 4 A of the main text). The networks built using the hallmark genes of WNT pathway using HuRI and Signor show different topologies. First, the network obtained via HuRi has been built with an input gene list that, after filtering, was bigger than the one used as input for Signor. Both databases can be interpreted as networks following a scale-free distribution. However, given the different topological constraints, (HuRi undirected, Signor directed) HuRi has in general a higher clustering coefficient because of dense modular clusters, while the hierarchical structure of Signor, typical of networks representing signaling pathways, is overall lower. This leads to a different number of edges available for the networks obtained using the different networks (347 edges with Signor and 260 with HuRi; 102 Nodes with Signor and 97 with HuRi). This observation

matches with HuRi's property of "small-world" network diameter that implies a consequent average shorter path between elements of the inferred subnetwork compared to the one inferred with Signor.

Starting genes for each group

WNT		SHH		Group3	Group4	
ALX4	HOXD13	ATOH1	PRKAR1A	ATM	ARID5B	MED12
APC	HOXD8	BBX	PTCH1	CRX	BARHL1	MYCN
ARID1A	HOXD9	BCOR	PTEN	GFI1B	BRCA2	OTX2
ARID2	IRX2	BRCA2	RARB	HLX	CDK6	POU2F1
ATM	IRX5	CEBPA	SMO	IRX6	CUX2	PRDM6
BAI3	LEF1	CREBBP	SOX13	KBTBD4	DLX5	SIX1
CSNK2B	LHX8	DDX3X	SOX2	LHX9	EN2	SIX6
CTNNB1	MAF	DMRTA1	SUFU	MYC	EOMES	TAL1
DDX3X	MSX2	EPHA7	TBX18	NKX3-2	FOXP2	TBR1
DLX3	NKX6-1	FBXW7	TCF4	PTEN	GFI1	UNCX
DLX4	ONECUT2	GLI2	TERT	SMARCA4	GFI1B	ZIC1
EMX2	OSR2	GSE1	TP53		KBTBD4	ZMYM3
EN1	PAX3	IDH1			KDM6A	
EPHA7	PIK3CA	KMT2C			KMT2C	
FOXF1	PITX1	KMT2D			KMT2D	
HOMEZ	PRRX1	NFATC1			LHX1	
HOXC4	RUNX2	PBX1			LHX2	
HOXC5	SHOX2	POU3F3			LHX5	
HOXD11	SYNCRIP	POU6F2			LMX1A	

Legend:
Blue: Genes present in both databases
Purple: Genes present just in Huri
Green: Genes present just in Signor
White: Genes not found in any database

Figure B. Initial genes selected per each group. Not all the selected genes were present in the database or managed to be connected with any paths of length minor or equal 4. The color in the table shows which genes were found in Huri, Signor, both or none of them.

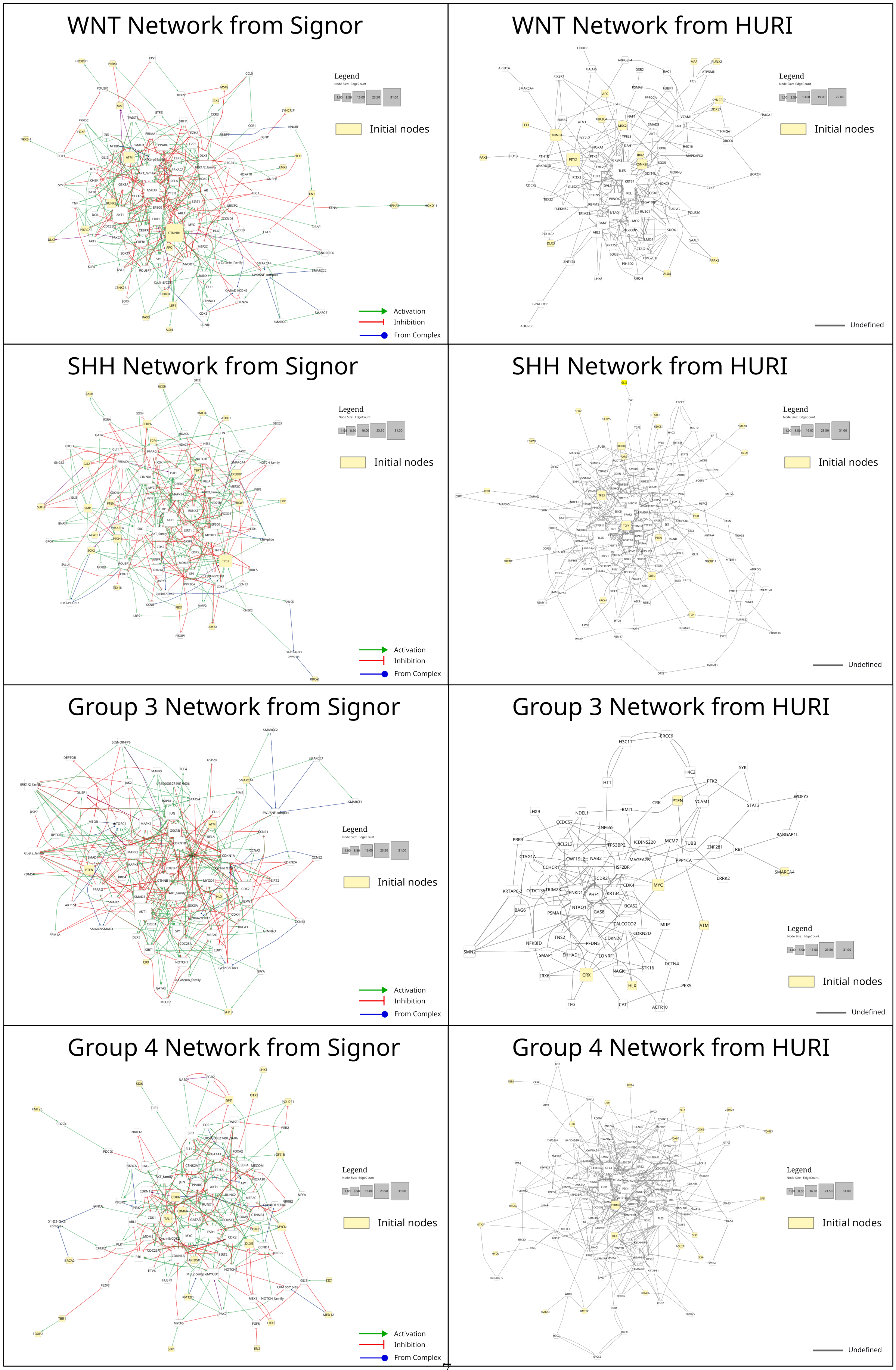

Figure C. Cytoscape visualization of each subgroup's network generated using Signor and HURI.

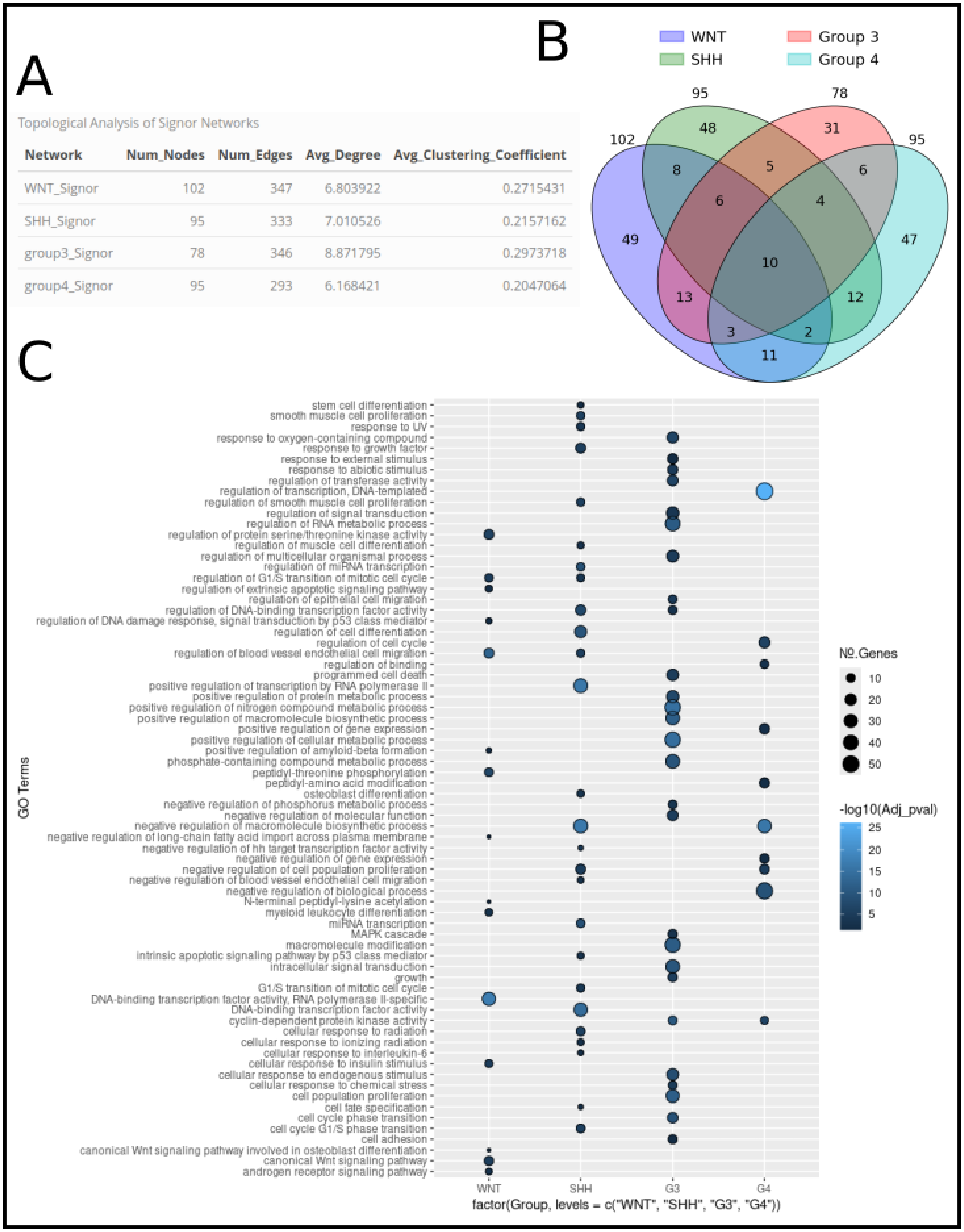

Topological Analysis of Signor Networks

Network	Num_Nodes	Num_Edges	Avg_Degree	Avg_Clustering_Coefficient
WNT_Signor	102	347	6.803922	0.2715431
SHH_Signor	95	333	7.010526	0.2157162
group3_Signor	78	346	8.871795	0.2973718
group4_Signor	95	293	6.168421	0.2047064

Figure D. Topological and functional comparison of the NeKo networks created for the medulloblastoma subgroups using the Signor databases. A) Comparison of the main topological networks properties between the four subgroups B) Venn diagram displaying the common nodes among each subgroups. C) Functional comparison of the four medulloblastoma subgroups network generated from the Signor database, using the ClueGO Cytoscape plug-in. Only leading terms with an adjusted p-value ≤ 0.05 are presented.

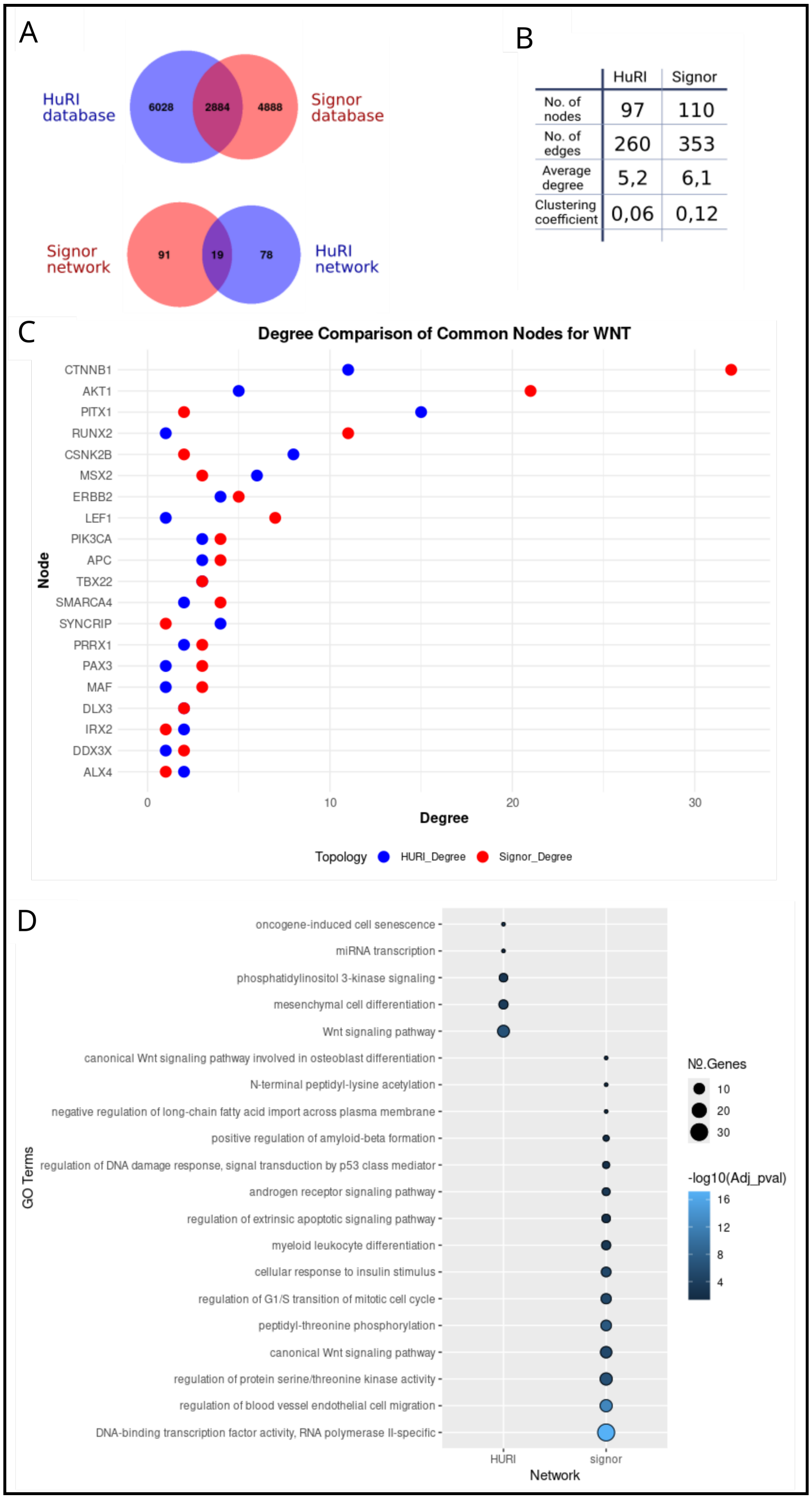

	HuRI	Signor
No. of nodes	97	110
No. of edges	260	353
Average degree	5,2	6,1
Clustering coefficient	0,06	0,12

Figure E. Topological and functional comparison of the NeKo networks created for the medulloblastoma subgroups using the Signor database, with a focus on the WNT subgroup. A) Comparison of the total number of nodes included in the HuRI and Signor databases (upper diagram) and the number of nodes included in their respective networks for the WNT subgroup. B) Comparison of the main topological networks properties between the two databases C) Comparison of the degree of the common nodes of the WNT subgroup networks built from HuRI and Signor. D) GO term overrepresentation comparison between the WNT subgroup networks built from Signor and HuRI.

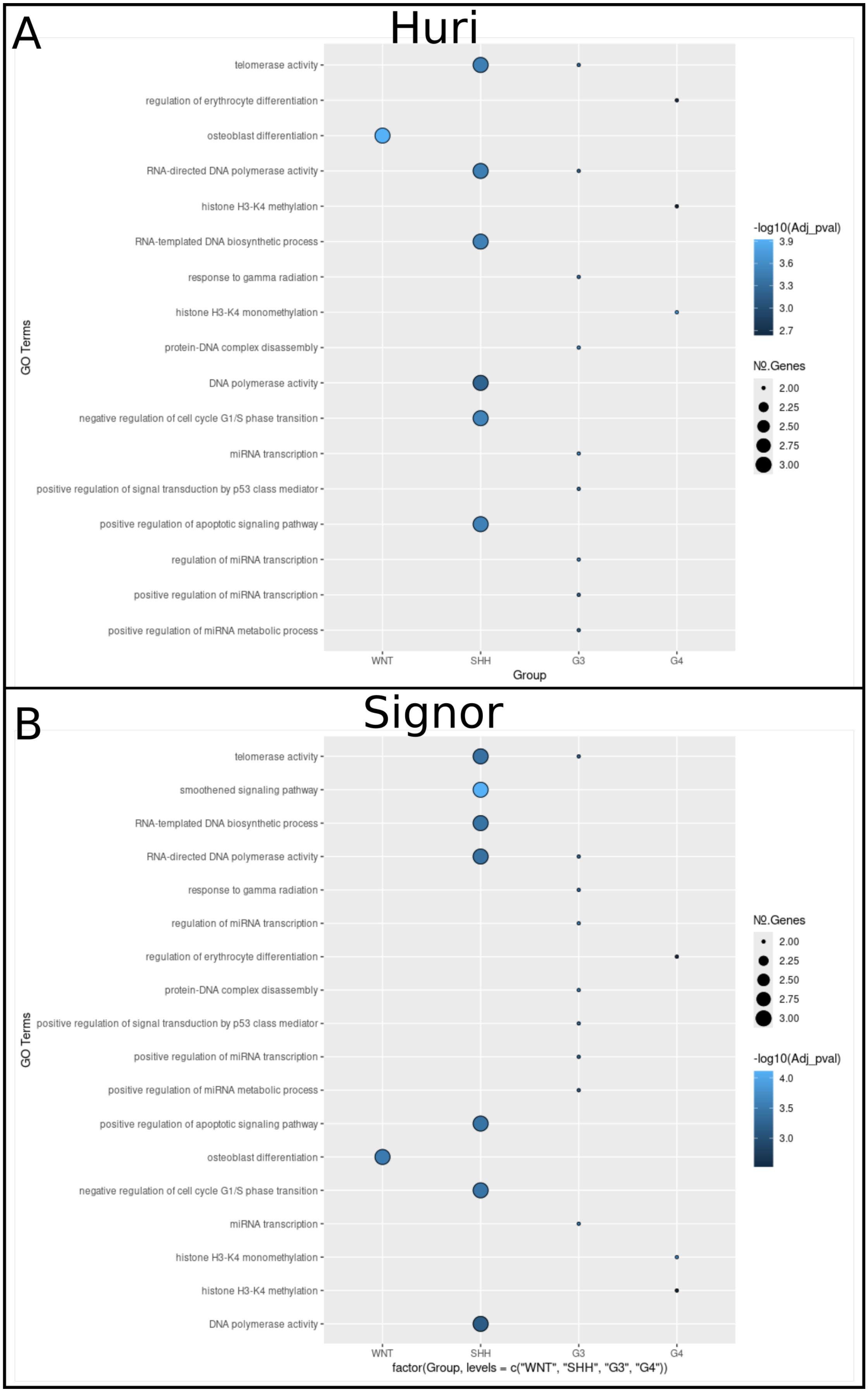

Figure F. GO term overrepresentation of the seeds genes present in A) the Huri database and B) the Signor 3 database.

S6 Additional analysis on Use case 2 - Topological differences between Flobak et al. and NeKo

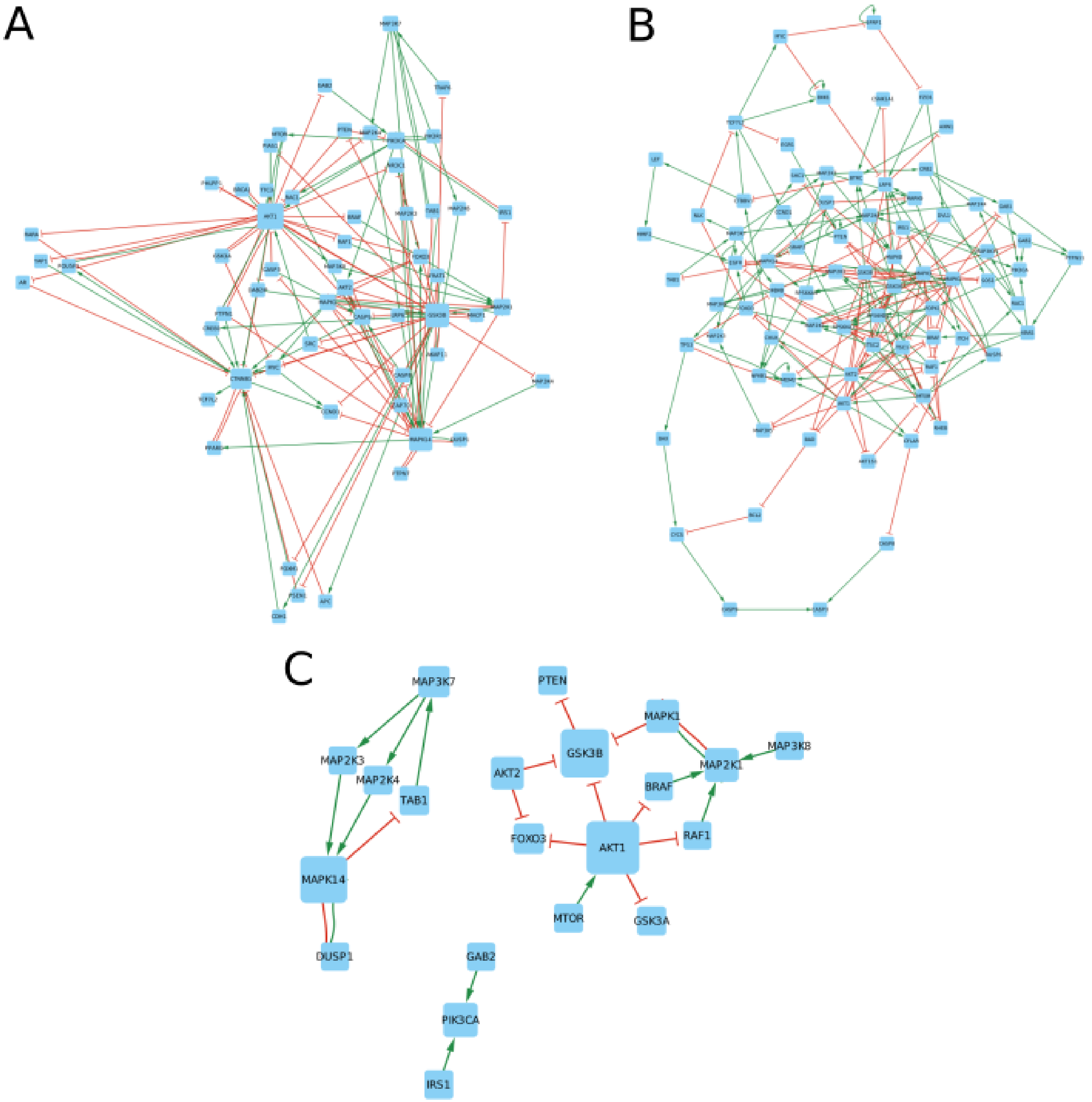

Figure G. A) Cytoscape visualization of the network obtained with NeKo B) Cytoscape visualization of the cascade network, C) common interactions between the two networks.

A

Topological Analysis of NeKo and Cascade Networks

Network	Num_Nodes	Num_Edges	Avg_Degree	Avg_Clustering_Coefficient
NeKo	57	140	4.912281	0.4283600
Cascade	77	217	5.636364	0.0681066

B

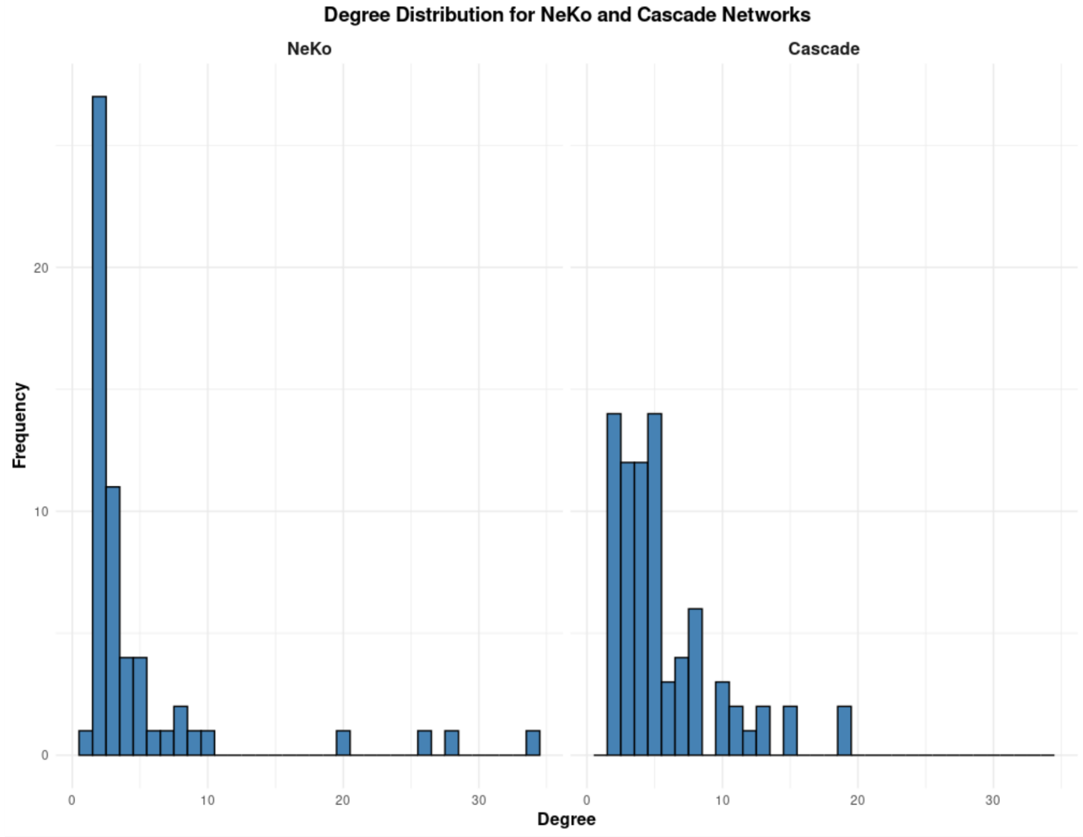

Figure H. A) Topological overview of the two networks, B) Degree distribution.

S7 Benchmark

We have performed a benchmark of the RPE strategy, the fastest approach in the NeKo package, which implements both DFS (Depth-First Search) and BFS (Breadth-First Search) algorithms, to evaluate the computational efficiency of NeKo. The benchmark assesses execution time across different initial gene sets (chosen randomly from Uniprot `https://www.uniprot.org/`), ranging from 40 to 260 initial genes of interest (specifically, 40, 76, 109, 148, 183, 223, and 260 genes). The "Post Translational" database of Omnipath has been used as Universe for this benchmark (134282 interactions).

The computational experiments were conducted on a Dell laptop equipped with a 12-core Intel i7-1365U processor and 16GB of RAM. The results, shown in Fig I, compare three different search strategies:

- DFS with maxlen = 3 (red)
- BFS with maxlen = 3 (blue)
- BFS without maxlen constraints (green)

As expected, DFS exhibits higher computational cost than BFS, as it explores a greater number of paths while maintaining a depth constraint. BFS with maxlen = 3 remains computationally efficient, scaling well across all gene set sizes. However, BFS without maxlen constraints shows an exponential increase in execution time as the network grows, demonstrating the importance of applying a max depth limit in large-scale analyses.

We also label each data point with the final number of nodes in the reconstructed network, highlighting the expansion effect of each search strategy. The results reinforce that applying constraints to BFS (maxlen = 3) provides a balanced trade-off between computational efficiency and network completeness.

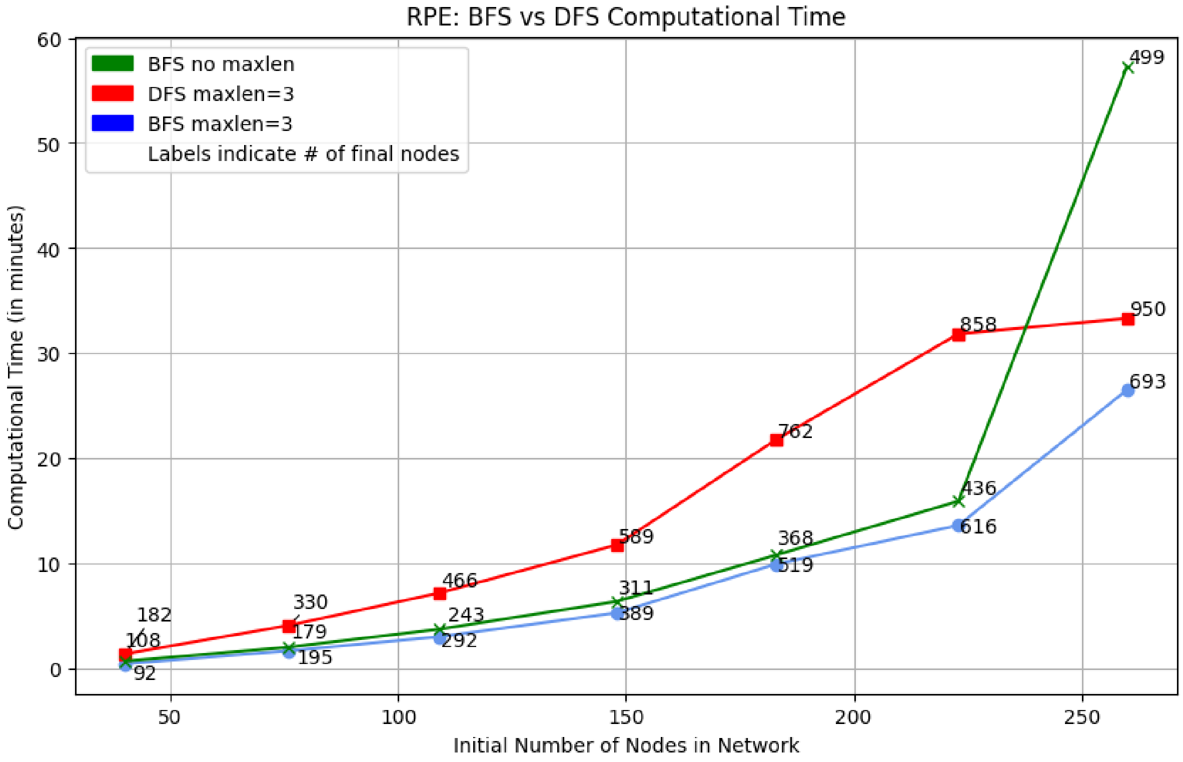

Figure I. Benchmark of the RPE strategy in NeKo, comparing computational time execution for DFS (maxlen = 3), BFS (maxlen = 3), and BFS without maxlen constraints. The x-axis represents the initial number of genes of interest, while the y-axis shows the execution time in minutes. Each data point is labeled with the final number of nodes in the reconstructed network, illustrating how network expansion impacts computation time. BFS without maxlen constraints (green) grows exponentially, while DFS (red) remains computationally demanding. BFS with maxlen = 3 (blue) offers an efficient trade-off between performance and completeness.

References

[1] Türei D, Korcsmáros T, Saez-Rodriguez J. OmniPath: guidelines and gateway for literature-curated signaling pathway resources. Nature Methods. 2016 Nov;13(12):966-7.

[2] Luck K, Kim DK, Lambourne L, Spirohn K, Begg BE, Bian W, et al. A reference map of the human binary protein interactome. Nature. 2020 Apr;580(7803):402-8. Available from: https://doi.org/10.1038/s41586-020-2188-x.

[3] Northcott PA, Korshunov A, Witt H, Hielscher T, Eberhart CG, Mack S, et al. Medulloblastoma Comprises Four Distinct Molecular Variants. Journal of Clinical Oncology. 2011 Apr;29(11):1408-14. Available from: https://www.ncbi.nlm.nih.gov/pmc/articles/PMC4874239/.

[4] Lin CY, Erkek S, Tong Y, Yin L, Federation AJ, Zapatka M, et al. Active medulloblastoma enhancers reveal subgroup-specific cellular origins. Nature. 2016 Feb;530(7588):57-62. Publisher: Nature Publishing Group. Available from: https://www.nature.com/articles/nature16546.

List of Figures

A Composition of the NeKo package. Each package module (represented by a folder) contains the classes that compose the package. The module "core" contains the `Network` class, while "inputs" contains the `Universe`. The "methods" module contains the `Connections` class. The "annotations", "visual" and "output" contain respectively the `Onthology`, `Visualizer`, and `Export` classes. 2

B Initial genes selected per each group. Not all the selected genes were present in the database or managed to be connected with any paths of length minor or equal 4. The color in the table shows which genes were found in Huri, Signor, both or none of them. . 6

C Cytoscape visualization of each subgroup's network generated using Signor and HURI. . 7

D Topological and functional comparison of the NeKo networks created for the medulloblastoma subgroups using the Signor databases. A) Comparison of the main topological networks properties between the four subgroups B) Venn diagram displaying the common nodes among each subgroups. C) Functional comparison of the four medulloblastoma subgroups network generated from the Signor database, using the ClueGO Cytoscape plug-in. Only leading terms with an adjusted p-value ≤ 0.05 are presented. 8

E Topological and functional comparison of the NeKo networks created for the medulloblastoma subgroups using the Signor database, with a focus on the WNT subgroup. A) Comparison of the total number of nodes included in the HuRI and Signor databases (upper diagram) and the number of nodes included in their respective networks for the WNT subgroup. B) Comparison of the main topological networks properties between the two databases C) Comparison of the degree of the common nodes of the WNT subgroup networks built from HuRI and Signor. D) GO term overrepresentation comparison between the WNT subgroup networks built from Signor and HuRI. 9

F GO term overrepresentation of the seeds genes present in A) the Huri database and B) the Signor 3 database. 10

G A) Cytoscape visualization of the network obtained with NeKo B) Cytoscape visualization of the cascade network, C) common interactions between the two networks. 11

H A) Topological overview of the two networks, B) Degree distribution. 12

I Benchmark of the RPE strategy in NeKo, comparing computational time execution for DFS (maxlen = 3), BFS (maxlen = 3), and BFS without maxlen constraints. The x-axis represents the initial number of genes of interest, while the y-axis shows the execution time in minutes. Each data point is labeled with the final number of nodes in the reconstructed network, illustrating how network expansion impacts computation time. BFS without maxlen constraints (green) grows exponentially, while DFS (red) remains computationally demanding. BFS with maxlen = 3 (blue) offers an efficient trade-off between performance and completeness. 13
