# Supplementary material for: NeKo: A tool for automatic network construction from prior knowledge
Source: PLoS Comput Biol. 2025 Sep 16;21(9):e1013300. doi: 10.1371/journal.pcbi.1013300 (PMC12456767; doi:10.1371/journal.pcbi.1013300)
Supplement: Attachment — Submitted filename: Response_to_reviewers.pdf [file pcbi.1013300.s003.pdf]

NeKo: answers to the reviewers and TODO

# Email:

Thank you for submitting your manuscript to PLOS Computational Biology. After careful consideration, we feel that it has merit but does not fully meet PLOS Computational Biology's publication criteria as it currently stands. Therefore, we invite you to submit a revised version of the manuscript that addresses the points raised during the review process.

Please submit your revised manuscript within 60 days Mar 08 2025 11:59PM. If you will need more time than this to complete your revisions, please reply to this message or contact the journal office at ploscompbiol@plos.org. When you're ready to submit your revision, log on to https://www.editorialmanager.com/pcompbiol/ and select the 'Submissions Needing Revision' folder to locate your manuscript file.

Please include the following items when submitting your revised manuscript:

* A rebuttal letter that responds to each point raised by the editor and reviewer(s). You should upload this letter as a separate file labeled 'Response to Reviewers'. This file does not need to include responses to formatting updates and technical items listed in the 'Journal Requirements' section below.
* A marked-up copy of your manuscript that highlights changes made to the original version. You should upload this as a separate file labeled 'Revised Manuscript with Track Changes'.
* An unmarked version of your revised paper without tracked changes. You should upload this as a separate file labeled 'Manuscript'.

If you would like to make changes to your financial disclosure, competing interests statement, or data availability statement, please make these updates within the submission form at the time of resubmission. Guidelines for resubmitting your figure files are available below the reviewer comments at the end of this letter

We look forward to receiving your revised manuscript.

Kind regards,

Alberto J M Martin, Ph.D.
Academic Editor
PLOS Computational Biology

Mark Alber
Section Editor
PLOS Computational Biology

Additional Editor Comments:

Please do address issues raised by reviewers, specially those regarding escalation on larger datasets, limitations and validation of the tool

Journal Requirements:

1) We ask that a manuscript source file is provided at Revision. Please upload your manuscript file as a .doc, .docx, .rtf or .tex. If you are providing a .tex file, please upload it under the item type u2018LaTeX Source Fileu2019 and leave your .pdf version as the item type u2018Manuscriptu2019.

2) Please provide an Author Summary. This should appear in your manuscript between the Abstract (if applicable) and the Introduction, and should be 150-200 words long. The aim should be to make your findings accessible to a wide audience that includes both scientists and non-scientists. Sample summaries can be found on our website under Submission Guidelines:
https://journals.plos.org/ploscompbiol/s/submission-guidelines#loc-parts-of-a-submission

3) Please upload all main figures as separate Figure files in .tif or .eps format. For more information about how to convert and format your figure files please see our guidelines:
https://journals.plos.org/ploscompbiol/s/figures

4) Please ensure that all Figure files have corresponding citations and legends within the manuscript. Currently, Figure 2 in your submission file inventory does not have an in-text citation. If the figure is no longer to be included as part of the submission, please remove it from the file inventory.

5) We have noticed that you have uploaded Supporting Information files, but you have not included a list of legends. Please add a full list of legends for your Supporting Information files after the references list.

6) Some material included in your submission may be copyrighted. According to PLOSu2019s copyright policy, authors who use figures or other material (e.g., graphics, clipart, maps) from another author or copyright holder must demonstrate or obtain permission to publish this material under the Creative Commons Attribution 4.0 International (CC BY 4.0) License used by PLOS journals. Please closely review the details of PLOSu2019s copyright requirements here: PLOS Licenses and Copyright. If you need to request permissions from a copyright holder, you may use PLOS's Copyright Content Permission form.
Please respond directly to this email and provide any known details concerning your material's license terms and permissions required for reuse, even if you have not yet obtained copyright permissions or are unsure of your material's copyright compatibility. Once you have responded and addressed all other outstanding technical requirements, you may resubmit your manuscript within Editorial Manager.
Potential Copyright Issues:

- The following Figure contains a logo or branding: Figure 1. We are not permitted to publish this under our CC-BY 4.0 license, even with permission. We ask that you please remove or replace it.

7) Please amend your detailed Financial Disclosure statement. This is published with the article. It must therefore be completed in full sentences and contain the exact wording you wish to be published.
- State the initials, alongside each funding source, of each author to receive each grant. For example: "This work was supported by the National Institutes of Health (####### to AM; ###### to CJ) and the National Science Foundation (###### to AM)."
- State what role the funders took in the study. If the funders had no role in your study, please state: "The funders had no role in study design, data collection and analysis, decision to publish, or preparation of the manuscript.".
If you did not receive any funding for this study, please simply state: u201cThe authors received no specific funding for this work.u201d

****

Dear Editor,

We would like to thank the reviewers for their careful reading of our manuscript and their constructive comments.

We address point by point below.

Reviewer #1:

The main contribution of this work is the development of NeKo, a Python package designed to automate the construction of biological networks using prior knowledge databases. By integrating molecular interactions from public databases and applying flexible connection strategies, NeKo simplifies the traditional manual and time-consuming process of network curation. This tool has two well defined aims, i) to conduct network analysis and ii) to build a predictive model. Depending on the chosen aim, the pipeline adapts accordingly. The paper demonstrates so through two use cases: 1. Medulloblastoma networks: NeKo reveals subgroup-specific mechanisms of disease progression. 2. Drug synergy modeling: NeKo generates biologically meaningful networks that can predict drug synergies, and their quality is only a bit lower than the manual curated one.

The development of this tool is significant for computational biology as it reduces the complexity of network biology reconstruction, it enhances accessibility offering flexible and user-friendly connection strategies, and it is compatibility with other tools so it can be included in different pipelines, for example, offering network annotation with Gene Ontology terms. Such advancements are crucial for promoting broader community research.

Finally, the authors provide detailed descriptions of NeKo's algorithms and workflow, accompanied by Jupyter notebooks and extensive documentation. The code is publicly

available at GitHub (https://github.com/sysbio-curie/Neko), and supporting data for case studies is also shared in supplementary materials.

I would like to conclude by expressing my appreciation for the paper. It is evident that the study is both well-organized and deeply grounded in thorough research.

Major suggestions:

- The authors present an automated approach for constructing networks, and since it is a novel implementation, I strongly suggest they highlight these novel aspects (e.g. GO enrichment analysis) by adding a table comparing NeKo with existing tools, for example, Augusta and NetworkCommons (which are mentioned in the text), and tools such as Pathway Commons, STRING, and more.

We thank the reviewer for this suggestion. We provide this table here, and in the README of the GitHub. This table contains some features and 3 other tools, but this table should be regularly updated, and extended as new tools or features emerge. This is why we choose to put it in the GitHub where it can be modified.

We do not intend to use NeKo as an alternative to many of these tools, but rather as a complement to them (depending on the question).

**Features**	**NeKo**	**Pathway-Commons**	**STRING**	**Augusta**	**Network-Commons**	**phuEGO**
Network Creation and Manipulation	Yes	No	Yes (limited)	Yes	Yes	Yes
Integration of Multiple Databases	Yes	Yes	Yes	No	Yes	No
Gene-to-Phenotype Mapping	Yes	No	No	No	No	No
Network Visualization	Yes	Yes	Yes	No	Yes	No
Boolean Network Inference	Yes	No	No	Yes	No	No
Data Download in Multiple Formats	Yes	Yes	Yes	No	No	No
Web-Based Access	No	Yes	Yes	No	No	No
Graph (Topology) Based Network Inference	Yes	No	No	No	Yes (limited)	No
Propagation Based Network Inference	No	No	No	No	Yes	Yes
ILP Based Network Inference	No	No	No	No	Yes	No
Network Contextualization Based on Genes of Interest	Yes	No	No	No	No	Yes

Network Contextualization Based on Omics Activity Signatures	No	No	No	No	Yes	No

- The authors presented the time it takes of the construction of small networks (<=50) and ~300 nodes networks, being seconds and up to two minutes, respectively. I have two comments to this. First, it would be useful to understand NeKo's performance and robustness on larger datasets (e.g. thousands of nodes). To this aim, I suggest mentioning it, implementing some kind of performance metrics such as computational time and edge accuracy, depending on the size of the network. Second, I suggest contextualizing the time with, for example, CPUs, RAM, OS, etc. or if the code automatically parallelize the tasks. I leave it to the authors to decide whether these specifications should be included in the main text or in the supplementary information.

To answer the reviewer's comment, we benchmarked the Reciprocate Pathway Extender (the fastest among all the strategies) with random genes from Uniprot. The results are summarized in the figure below and in the supplementary material.

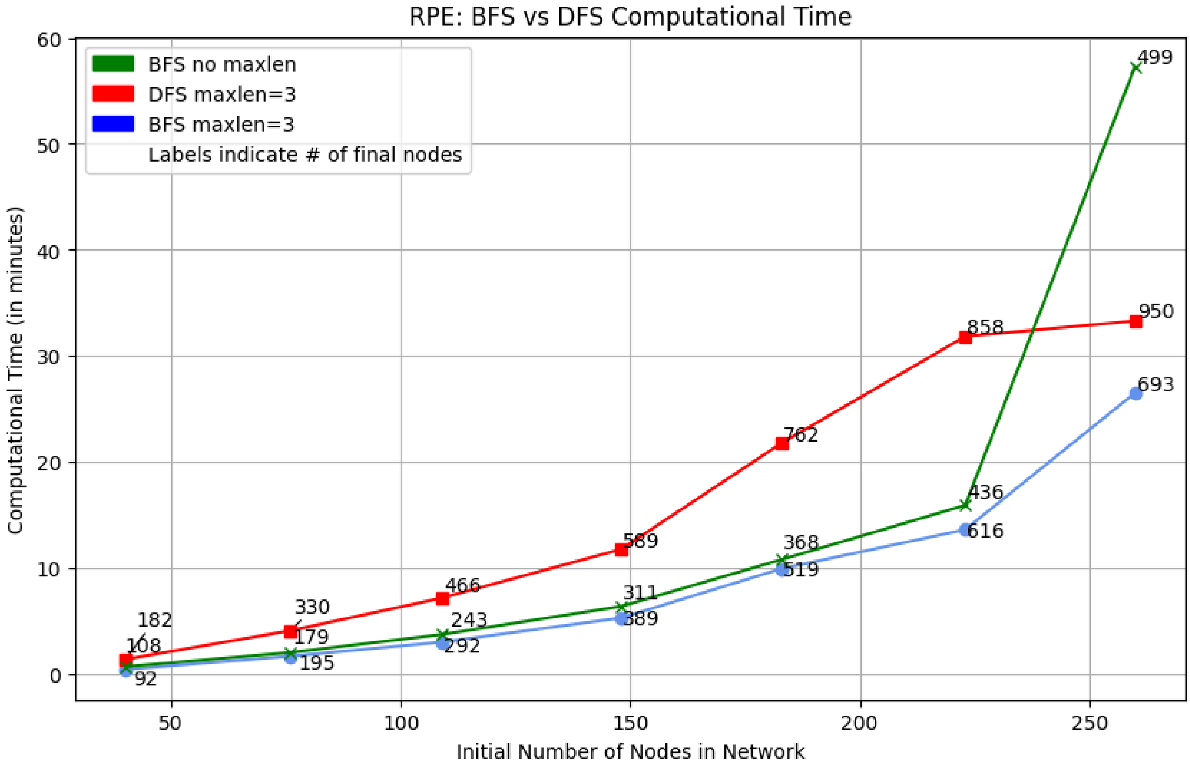

BFS with maximum length (max len = 3) performs better than DFS with maximum length = 3. Interestingly enough, when using BFS with no maximum length, the computational time is greater than when using DFS when the number of initial nodes is very high. This is because BFS explores the database network widely instead of

deeply, causing many more recursions. Apart from this, BFS performs better than DFS because it returns just the very first shortest path it finds between each pair of nodes. The user can always choose to constrain the research in order to avoid looking across the whole database, reducing computational time.

We would like to clarify that in the main text, we mention: “The methods presented here complete almost instantly when building small networks (50 nodes), while above 300 nodes the process may take up to two minutes”. In this case, we refer to the *final* number of nodes in the network and *not* the *initial* one.

This benchmark has been performed on a Dell laptop with a 12-core i7 1365U processor with 16Gb of RAM. We have added a new section in the supplementary material (Section 7 of “Additional Information”) with more insights from the benchmark.

- Could this tool be used for different organisms other than human, mouse or rat, for example, linking it to different databases? It could be useful to specify the organism. I would recommend to the authors to add a brief explanation about this in the main text.

  Currently networks only for human, mouse and rat are available in the built-in resources (SIGNOR, PhosphoSitePlus, OmniPath) . We intend to improve this part and extend the possibility of translating identifiers for other organisms.

- In the Supplementary Material, the authors organized the inputs, the scripts and the outputs in different folders. This is done for both use cases. Despite the fact each file has a description, I suggest implementing an explicit tutorial using these data, specifying which script to use first with which input. This would make the learning process much faster.

  We have updated the Supplementary Materials on GitHub to the latest version. Moreover, we have added in each README inside each Use Case folder, additional information on which Notebooks to run and how. All the code in the notebook runs automatically and there is no need to modify the code or update the paths that have been set. We have already provided partial output in the *Figure* folder and *SIF* folder in case the user wants to skip running the notebooks and directly analyze the output. Nevertheless, we invite the users to explore all the notebooks to get familiar with the structure of NeKo.

Minor suggestions:

- P4. Where is the function find_upstream_cascades? Is this the find_paths method from the print_my_paths function? I

The function “find_upstream_cascades” is implemented in the Connections class which is used to store the base algorithms that the Network class leverages in the strategies. In particular, the strategy that uses the “find_upstream_cascades” function is “connect_to_upstream_nodes”.

- P4. In the last paragraph, line four, it says eah instead of each.

This has been fixed.

- The first time SIF is mentioned as Simple Interaction Format is in page 6. I suggest putting it before, for example in page 4.

This has been fixed.

- I suggest that in the caption of Fig. 2 the authors specify that the Resources DataFrame is indeed the Universe DataFrame. As well as to include the word “figure” before the 2 in section 2.3.2 (page 5).

We have modified both the figure and the caption to make it coherent with the text.

- P5. The second-to-last phrase of section 2.3.2 should be “The better support of these databases was also motivated by the significance of their large…”. However, the authors could rephrase it to make it clearer.

We replaced this sentence by: “We chose to include support for these databases because of their high quality.”

- P8. When describing the Module Connection Mapper algorithm, the authors explain that there can be three modes of connections: IN, OUT and BOTH. However, in figure 3 it says ALL instead of BOTH. I suggest using the same mode name.

We have modified BOTH to ALL to use the same name all over the text

- P9. At the end of the last paragraph (section 3.1) it is written “(REF)”. I am assuming the authors forgot to add this reference(s), thus I suggest adding it/them.

We included the proper reference.

- I suggest increasing the font size of axis ticks and labels, as well as the quality of Fig. 5 (especially 5C). Check the supplementary figures as well.

We have updated the figure accordingly.

- How much does the result would change when using different inputs of the same network?

This is a tricky question because it depends on how far the initial nodes are and which strategy is chosen to build the network.

Let us assume that the user starts with 4 genes and builds a network using the RPE strategy. The result can be a fully connected network of 20 nodes. If among these 20 genes, we would randomly select 4 genes, and once again build a network from these 4 genes, the final result will be different. There are two reasons. the first one is that these 4 random genes may be far from each other and the way to connect them would include different genes. The second one is that depending on the strategy chosen, the search will select different genes from the database: the RPE chooses the shortest path and the INE includes all the possible paths, for example.

Reviewer #2:

The manuscript "NeKo: a tool for automatic network construction from prior knowledge" provides valuable insights into biological networks in the filed of transcriptomics. The authors have done excellent work in developing a Python package for construction of biological networks by integrating molecular interactions from various databases. The study aims to develop a Python package for construction of molecular network and also model drug synergies. The tool is available in github. However, the following issues need to be addressed before publication.

1) Which databases are integrated for genes and drugs to be addressed more specifically.

We have extended the manuscript slightly to address the reviewer comment:

From: “By default, NeKo derives interactions from OmniPath (11), but it can be set to use other public or proprietary databases.”

To: “By default, NeKo derives interactions from OmniPath (11), which allows extractions of interactions from multiple sources, and NeKo can also be configured to use other public or proprietary databases. We expect that the reader will access the OmniPath manuscript for additional information on sources covered. We also added simple direct clients to SIGNOR and PhosphoSitePlus because of their frequent updates and high significance in the field.

2) Comparison with similar packages and its advances to be highlighted.

We addressed this question with a table comparing a list of features in 4 packages (Table in the GitHub and response to Reviewer 1).

3) Limitations of NeKo are to listed.

We have listed some of the limitations of NeKo in the discussions but we have extended on it with this paragraph below.

“Another limitation resides in the Boolean models that can be built with NeKo. The heuristic approach used to shape the Boolean equations that characterize each node of the network does not lead to any significant attractor in most cases. This is because NeKo does not rely on data to support or constrain specific network states, meaning that the attractors reached when simulating the Boolean model often do not have biological relevance. The main issue arises from the large number of edges introduced into the network, which generates excessive noise and reduces the interpretability of the model. Future improvements could involve refining the edge selection process to reduce noise and improve the meaningfulness of attractors. Finally, the graphic library used to visualize the network, Graphviz, struggles with performance and readability when dealing with a high number of nodes in Jupyter notebooks. We plan in future updates to support multiple libraries for graph visualization, such as Igraph or NetworkX, to provide more efficient and interactive alternatives.}

”

Reviewer #3:

Review of “NeKo: a tool for automatic network construction from prior knowledge”

The manuscript introduces NeKo, a Python-based tool designed to automate the construction of biological networks by integrating molecular interactions from multiple prior knowledge databases. The authors aim to reduce the time-consuming and labor-intensive manual curation traditionally required in knowledge-driven network construction while providing flexibility. The manuscript highlights two use cases: constructing a network for medulloblastoma subgroup-specific signatures and modeling drug synergies using logical models.
NeKo represents a significant step forward in streamlining network construction and demonstrates the potential for broad application in systems biology. However, several major and minor issues need to be addressed to enhance the manuscript's clarity, rigor, and utility.

Major Comments

1. The authors provide a solid section on strategy selection, including descriptions of available algorithms and their applications. However, as strategy selection is a key challenge for usability, this section could be broadened to include more detailed guidance, examples, or decision trees to assist users in selecting the optimal strategy for their specific research questions. Clearer recommendations would improve accessibility for non-expert users and reduce the learning curve.

The choices for the strategy are very dependent on the questions, which makes it very hard to provide guidance. However, we provide more insights on how to use the strategies in concrete examples in the documentation (Jupyter notebooks #3, #6, and #9).

2. NeKo’s model for predicting drug synergies performed relatively poorly compared to the manually curated model. The manuscript should discuss whether the chosen strategy was optimal for this task and provide insights into how strategy selection influences performance. Was the poor result due to inherent limitations in the method or suboptimal strategy alignment?

We find it important to emphasize that we do not claim an automatically generated network can surpass the quality of a manually curated one. Rather, we argue that it provides a reasonable starting point, from which manual modifications can be made to enhance performance. The automatically constructed model indeed performed worse than the meticulously and manually curated model, which was expected. We aimed to demonstrate that it is possible to generate a functional draft model with minimal effort, which can then serve as a foundation for further manual refinement and optimization.

3. While the manuscript discusses NeKo's integration with databases like OmniPath and SIGNOR, a systematic evaluation of how database choice impacts network quality is important. This is particularly substantial given NeKo's reliance on external resources, and the lack of such benchmarking may limit confidence in its results.

The choice of database depends on many factors that are specific to the research topic: if the proteins of interest are not sufficiently covered by the largest and most up to date causal network SIGNOR, dozens of additional resources can be queried and combined together from OmniPath. For example, PhosphoSitePlus, phospho.ELM, and some others cover kinase cascades, many databases, such as MatrixDB or CellPhoneDB cover extracellular and ligand-receptor interactions. This diversity also foreshadows that a fair and meaningful benchmark of network databases would be a complex task, a topic for a whole other research article. On the other hand, if the pathway of interest is well covered by certain resources, the unnecessary addition of more resources can increase the noise and result in worse outputs.

Still to answer the reviewer's concern, we have already tested with the same list of genes and interrogated different databases for the Medulloblastoma example. We show that using the same strategy the networks are different but they provide complementary information with the focus that each database has. The results can be found in section 5 in the supplementary material for comparison between HURI and SIGNOR, and in the GitHub for Omnipath and SIGNOR: https://github.com/sysbio-curie/NeKo_Supp_Mat/tree/main/Use_case1/Northcott_et_al/notebooks).

4. While NeKo is tested on small-to-medium networks, its performance on larger datasets (>1000 nodes) is not discussed. Computational benchmarks assessing runtime and memory usage across varying network sizes would be helpful, especially for researchers handling large omics datasets.

We have performed a benchmark using the "Post Translational" database from OmniPath (134,282 interactions) and run the Reciprocate Pathways Extender strategy to build networks of different sizes with different algorithms (BFS and DFS). The whole analysis is reported in the supplementary material "Additional Information", in Section 7. We have performed a benchmark using the "Post Translational" database from OmniPath (134,282 interactions) and run the Reciprocate Pathways Extender strategy to build networks of different sizes with different algorithms (BFS and DFS). The whole analysis is reported in the supplementary material "Additional Information", in Section 7.

5. Comparing NeKo's functionality and outputs with widely used alternatives, such as STRING or Ingenuity Pathway Analysis (IPA), would provide context for its capabilities. Highlighting NeKo's strengths (e.g., flexibility, export formats) and limitations relative to these tools would better position it within the field of network construction.

We discuss the comparison between NeKo and similar existing software in the response to comment #1 from Reviewer #1, where we also point to a new table that we added to our GitHub repository. STRING is a network database, which is not an alternative, but a potential input for NeKo. However, because it is a large collection of undirected interactions, without causality information, it is not particularly well suited to NeKo's typical applications. Ingenuity Pathway Analysis, in contrast to all software discussed in the manuscript and the responses here, is not a free software: its license costs about 32,000 USD/year, which makes it practically unavailable for the academic community. As such, despite having some overlap with NeKo in features and purpose, we decided not to include it in the comparison.

Minor Comments

- The manually curated network's AUC value is not reported in Figure 5, making it difficult to assess the performance gap between NeKo-derived and curated networks. This metric should be included for a complete comparison.

We have recreated plots to include the AUC in both figures. There is indeed a performance gap, and the use case is meant to demonstrate how quickly a starting point for further manual curation can be generated.

- On page 4, the text mentions "activation" edges, while Figure 2 refers to "stimulation."

This has been fixed

- On page 9, a citation appears as "(REF)." This needs correction.

This has been fixed.

- On page 10, "Addittional" contains a typographical error.

This has been fixed.
