# Supplementary material for: NeKo: A tool for automatic network construction from prior knowledge
Source: PLoS Comput Biol. 2025 Sep 16;21(9):e1013300. doi: 10.1371/journal.pcbi.1013300 (PMC12456767; doi:10.1371/journal.pcbi.1013300)
Supplement: Attachment — Submitted filename: Answer_to_review_final.pdf [file pcbi.1013300.s004.pdf]

Dear Editor,

We would like to thank the reviewer for this last comment on our manuscript. We hope that our answer will satisfy the concern raised by the reviewer. You will find below our response.

Best regards,

Marco Ruscone and Laurence Calzone, on behalf of all co-authors

****

Review #3

Thank you for addressing my comments and providing the comparative table in your GitHub repository.

However, I still have concerns regarding the exclusion of Ingenuity Pathway Analysis (IPA) based solely on its licensing cost. Given IPA's frequent citation and feature overlap with NeKo, a brief qualitative comparison could strengthen your manuscript and clarify NeKo's unique contributions.

I suggest briefly discussing IPA's distinguishing features and contexts compared to NeKo, despite the cost limitation.

To address the concerns, we have requested trial versions since none of our institutes hold a license, but we encountered various issues with installation.
Moreover, to promote open science, we believe that only open-source software should be used to evaluate and benchmark the features of the tools of the community.
Still, to address the reviewer's comments, we summarized IPA's features from what was reported in publications and online tutorials to qualitatively compare IPA with NeKo's functionalities.

The results have been summarized in the table below. This table will be included as supplementary material on the GitHub page [link].

Most of the differences stem from the fact that Ingenuity includes data analysis features that NeKo does not. This table emphasizes one of NeKo's main goals, which is to complement features from other tools and open resources, such as NetworkCommons, as mentioned in the article, and facilitate the construction of networks from prior knowledge. In summary, NeKo could and should be used in combination with other software for a full analysis.

Type of inputs	Feature	Description of the feature	Overlap with NeKo
**Data**	**Canonical Pathway Analysis**	Detect which signalling or metabolic	No

		pathways are statistically enriched in the data and predict their activation or inhibition states.	
	**Upstream Regulator Analysis**	Identify transcription factors, miRNAs, cytokines, kinases, or drugs that may be driving the observed expression changes.	Partial:<br>Upstream regulators can be selected with NeKo and filtered with TFs
	**Downstream Effects Analysis**	Predict which biological functions, diseases, or phenotypes are likely to be altered as a consequence of the expression profile.	No
	**Comparison Analysis**	Compare multiple experiments (time-points, doses, tissues, treatments) in a heatmap, clustering pathways, regulators, and functions across conditions.	No
	**Broad multi-omics & batch support**	Accepts RNA-seq, proteomics, metabolomics, single-cell, microarray, SNP, and small bespoke gene/chemical lists, with plug-ins for CLC and count-matrix uploads.	No
	**IsoProfiler & Isoform View**	Drill down to transcript-isoform level, spot isoform switching, and leverage GTEx tissue-specific expression to prioritise variants.	No
**Data + Database**	**Phosphorylation Analysis**	Interpret phospho-proteomics datasets, uncovering regulators and signalling pathways behind phosphorylation	Yes<br>NeKo allows the user to explore phospho-proteomics databases such as Phosphosite Plus.

		changes.	
	**microRNA Target Filter**	Filter and visualise experimentally validated or predicted miRNA–mRNA interactions in the context of your expression data.	No
	**Causal Network Analysis**	Extend the upstream search into multi-layer causal networks to reveal higher-order regulators that explain the dataset. Search can be done in databases and inferred from data.	Partial:<br>NeKo searches upstream regulators only in annotated databases.
	**Toxicity Functions & Lists**	Link gene or compound data to clinical pathology end-points for mechanism-of-toxicity and safety-risk assessments.	No
**Database (IPA)**	**Analysis Match**	Automatically match the results against > 50000 public IPA analyses to find studies with similar or opposite biology.	No
	**Ingenuity Knowledge Base**	All analyses run against a deeply curated corpus of >8 million causal relationships derived from the literature, ensuring biological accuracy.	No
**Database / Network**	**Mechanistic Networks**	Generate plausible signalling cascades linking upstream regulators to downstream genes, offering hypotheses of the mode of action.	Yes<br>NeKo can generate signalling pathways linking upstream regulators to downstream genes that need to be specified by the user.
	**Regulator Effects**	Integrate Upstream and Downstream	Yes<br>NeKo allows the

		results to build causal narratives that link predicted regulators to functional outcomes.	merging and integration of Upstream and Downstream results to connect networks to phenotypes inferred from GO.
	**Interactive Network Analysis & Path Designer**	Build and edit gene, protein, miRNA, or chemical interaction networks and export publication-quality graphics.	Yes<br>All networks produced by NeKo can be manually modified and exported in different formats, as well as being visualized using the Graphviz package.
	**BioProfiler**	Rapidly profile a disease or phenotype to discover causally relevant genes, drugs, biomarkers, and toxicity targets.	No
**Network**	**Molecule Activity Predictor (MAP)**	“Paint” up- or down-regulation onto a pathway or sub-network and simulate the propagated effects to generate testable hypotheses.	Yes<br>NeKo allows the user to directly visualize the up- or down-regulations in the network, applying color-based filters to the graph.
	**AI-powered IPA Interpret**	An optional 2024 addition that uses natural-language summaries and shareable web reports to speed up interpretation and collaboration.	No
