# Supplementary material for: NeKo: A tool for automatic network construction from prior knowledge
Source: PLoS Comput Biol. 2025 Sep 16;21(9):e1013300. doi: 10.1371/journal.pcbi.1013300 (PMC12456767; doi:10.1371/journal.pcbi.1013300)
Supplement: Attachment — Submitted filename: Review.docx [file pcbi.1013300.s002.docx]

The main contribution of this work is the development of NeKo, a Python package designed to automate the construction of biological networks using prior knowledge databases. By integrating molecular interactions from public databases and applying flexible connection strategies, NeKo simplifies the traditional manual and time-consuming process of network curation. This tool has two well defined aims, i) to conduct network analysis and ii) to build a predictive model. Depending on the chosen aim, the pipeline adapts accordingly. The paper demonstrates so through two use cases: 1. Medulloblastoma networks: NeKo reveals subgroup-specific mechanisms of disease progression. 2. Drug synergy modeling: NeKo generates biologically meaningful networks that can predict drug synergies, and their quality is only a bit lower than the manual curated one.

The development of this tool is significant for computational biology as it reduces the complexity of network biology reconstruction, it enhances accessibility offering flexible and user-friendly connection strategies, and it is compatibility with other tools so it can be included in different pipelines, for example, offering network annotation with Gene Ontology terms. Such advancements are crucial for promoting broader community research.

Finally, the authors provide detailed descriptions of NeKo’s algorithms and workflow, accompanied by Jupyter notebooks and extensive documentation. The code is publicly available at GitHub (<https://github.com/sysbio-curie/Neko>), and supporting data for case studies is also shared in supplementary materials.

I would like to conclude by expressing my appreciation for the paper. It is evident that the study is both well-organized and deeply grounded in thorough research.

Major suggestions:

- The authors present an automated approach for constructing networks, and since it is a novel implementation, I strongly suggest they highlight these novel aspects (e.g. GO enrichment analysis) by adding a table comparing NeKo with existing tools, for example, Augusta and NetworkCommons (which are mentioned in the text), and tools such as Pathway Commons, STRING, and more.
- The authors presented the time it takes of the construction of small networks (<=50) and ~300 nodes networks, being seconds and up to two minutes, respectively. I have two comments to this. First, it would be useful to understand NeKo’s performance and robustness on larger datasets (e.g. thousands of nodes). To this aim, I suggest mentioning it, implementing some kind of performance metrics such as computational time and edge accuracy, depending on the size of the network. Second, I suggest contextualizing the time with, for example, CPUs, RAM, OS, etc. or if the code automatically parallelize the tasks. I leave it to the authors to decide whether these specifications should be included in the main text or in the supplementary information.
- Could this tool be used for different organisms other than human, mouse or rat, for example, linking it to different databases? It could be useful to specify the organism. I would recommend to the authors to add a brief explanation about this in the main text.
- In the Supplementary Material, the authors organized the inputs, the scripts and the outputs in different folders. This is done for both use cases. Despite the fact each file has a description, I suggest implementing an explicit tutorial using these data, specifying which script to use first with which input. This would make the learning process much faster.

Minor suggestions:

- P4. Where is the function find_upstream_cascades? Is this the find_paths method from the print_my_paths function? I
- P4. In the last paragraph, line four, it says eah instead of each.
- The first time SIF is mentioned as Simple Interaction Format is in page 6. I suggest putting it before, for example in page 4.
- I suggest that in the caption of Fig. 2 the authors specify that the Resources DataFrame is indeed the Universe DataFrame. As well as to include the word “figure” before the 2 in section 2.3.2 (page 5).
- P5. The second-to-last phrase of section 2.3.2 should be “The better support of these databases was also motivated by the significance of their large…”. However, the authors could rephrase it to make it clearer.
- P8. When describing the Module Connection Mapper algorithm, the authors explain that there can be three modes of connections: IN, OUT and BOTH. However, in figure 3 it says ALL instead of BOTH. I suggest using the same mode name.
- P9. At the end of the last paragraph (section 3.1) it is written “(REF)”. I am assuming the authors forgot to add this reference(s), thus I suggest adding it/them.
- I suggest increasing the font size of axis ticks and labels, as well as the quality of Fig. 5 (especially 5C). Check the supplementary figures as well.
- How much does the result would change when using different inputs of the same network?
